# Binary Metabolic Phenotypes and Phenotype Diversity Metrics for the Functional Characterization of Microbial Communities

**DOI:** 10.3389/fmicb.2021.653314

**Published:** 2021-05-25

**Authors:** Stanislav N. Iablokov, Pavel S. Novichkov, Andrei L. Osterman, Dmitry A. Rodionov

**Affiliations:** ^1^A.A. Kharkevich Institute for Information Transmission Problems, Russian Academy of Sciences, Moscow, Russia; ^2^PhenoBiome Inc., Walnut Creek, CA, United States; ^3^Sanford Burnham Prebys Medical Discovery Institute, La Jolla, CA, United States

**Keywords:** predictive functional profiling, metagenomic, 16S rRNA sequencing, metabolic phenotypes, microbiome, phenotype diversity

## Abstract

The profiling of 16S rRNA revolutionized the exploration of microbiomes, allowing to describe community composition by enumerating relevant taxa and their abundances. However, taxonomic profiles alone lack interpretability in terms of bacterial metabolism, and their translation into functional characteristics of microbiomes is a challenging task. This bottom-up approach minimally requires a reference collection of major metabolic traits deduced from the complete genomes of individual organisms, an accurate method of projecting these traits from a reference collection to the analyzed amplicon sequence variants (ASVs), and, ultimately, an approach to a microbiome-wide aggregation of predicted individual traits into physiologically relevant cumulative metrics to characterize and compare multiple microbiome samples. In this study, we extended a previously introduced computational approach for the functional profiling of complex microbial communities, which is based on the concept of *binary metabolic phenotypes* encoding the presence (“1”) or absence (“0”) of various measurable physiological properties in individual organisms that are termed phenotype carriers or non-carriers, respectively. Derived from complete genomes via metabolic reconstruction, binary phenotypes provide a foundation for the prediction of functional traits for each ASV identified in a microbiome sample. Here, we introduced three distinct mapping schemes for a microbiome-wide phenotype prediction and assessed their accuracy on the 16S datasets of mock bacterial communities representing human gut microbiome (HGM) as well as on two large HGM datasets, the American Gut Project and the UK twins study. The 16S sequence-based scheme yielded a more accurate phenotype predictions, while the taxonomy-based schemes demonstrated a reasonable performance to warrant their application for other types of input data (e.g., from shotgun metagenomics or qPCR). In addition to the abundance-weighted Community Phenotype Indices (CPIs) reflecting the fractional representation of various phenotype carriers in microbiome samples, we employ metrics capturing the diversity of phenotype carriers, Phenotype Alpha Diversity (PAD) and Phenotype Beta Diversity (PBD). In combination with CPI, PAD allows to classify the robustness of metabolic phenotypes by their anticipated stability in the face of potential environmental perturbations. PBD provides a promising approach for detecting the metabolic features potentially contributing to disease-associated metabolic traits as illustrated by a comparative analysis of HGM samples from healthy and Crohn’s disease cohorts.

## Introduction

Microbial communities are known to colonize various habitats, such as water, soil, and higher organisms including humans. Understanding various types of interactions within microbial communities (or microbiomes) and with an environmental niche is of obvious fundamental and practical importance, especially in the field of human health and disease. Thus, the problems of development and homeostasis of the human gut microbiome (HGM) attracted the most attention from many research groups due to many established associations between dysbiosis and various pathological conditions such as obesity (Cani and Van Hul, 2020), diabetes (Gurung et al., 2020), cancer (Guven et al., 2020), inflammatory bowel diseases (IBD) (Caruso et al., 2020), and neurological disorders (Grochowska et al., 2019). Additional tentative associations of HGM and human health include the regulation of blood pressure (Marques et al., 2018), neurodevelopment (Borre et al., 2014), bile acid metabolism (Ramirez-Perez et al., 2017), and immune homeostasis (Wang et al., 2019). The knowledge of HGM taxonomic composition and functional profiles would enable the identification of relevant features associated with such conditions, opening new diagnostic and therapeutic opportunities.

Rapid advancement of genomic technology enabled a comprehensive coverage of HGM by thousands of completely sequenced reference genomes (O’Leary et al., 2016; Forster et al., 2019; Poyet et al., 2019; Zou et al., 2019). Likewise, massive amounts of fecal samples from a variety of clinical and population studies have been taxonomically profiled by amplicon (16S rDNA) sequencing methodology. A comparative and correlative analysis of these taxonomic profiles versus various types of clinical and other metadata provides new powerful approaches toward the diagnostic and rational selection of probiotics. However, taxonomic profiles alone, no matter how useful for certain practical tasks, lack interpretability in terms of functional, most importantly metabolic, properties and interactions of microbial communities. A genome-based reconstruction of bacterial metabolic pathways and networks (Rodionov et al., 2010; Ravcheev et al., 2013; Khoroshkin et al., 2016; Romine et al., 2017) is a well-established methodology enabling a predictive metabolic modeling (Zhang et al., 2009; Pan and Reed, 2018). However, the extension of this methodology toward complex and widely variable microbial communities, despite some first encouraging steps (Zhang and Reed, 2014; Magnusdottir et al., 2017), represents a substantial challenge. To establish a reliable quantitative description (let alone mathematical model) of the metabolic potential of a microbial community from the 16S amplicon sequencing data using a bottom-up approach (from genome-wide to microbiome-wide metabolic reconstruction), we need to successfully address at least three critical issues.

First, a scalable functional profiling approach should adopt a standard language, i.e., a set of functional traits that can be confidently deduced from the genomes of individual species reflecting their measurable physiological, biochemical, or other properties. These traits (or rather presence and absence thereof) assigned to each genome in a representative collection of reference genomes would provide a foundation for converting the 16S rRNA gene profiles to functional profiles of microbial communities. The commonly used state-of-the-art tools, e.g., PICRUSt2 (Douglas et al., 2020) and Tax4Fun2 (Wemheuer et al., 2020), report the abundance of either gene families or automatically assigned metabolic pathways, deriving reference data from existing genomic databases of biochemical pathways such as the KEGG (Kanehisa et al., 2012). Previously, we introduced a different approach to the predictive metabolic profiling of microbial communities based on the concept of *binary metabolic phenotypes* (Rodionov et al., 2019), which are deduced from the complete genomes of reference bacteria using a subsystems-based metabolic reconstruction (Overbeek et al., 2005). Binary phenotypes represent measurable physiological properties (traits) of individual species such as the ability or inability to produce or consume certain metabolite or nutrient and encoded as “1” for a particular phenotype’s carriers and as “0” for non-carriers. The main distinctive aspect of this approach [originally introduced and described for the example of predicted B-vitamin prototrophy and auxotrophy (Rodionov et al., 2019)] is in aggregating data on reconstructed and curated metabolic pathways into a single binary (1 or 0) encoding of a particular functional trait, which has a straightforward biological interpretation.

A second challenge of genomic reference-based functional profiling is the limited coverage of the HGM species by complete reference genomes (amenable to metabolic reconstruction) exacerbated by an even more limited precision of 16S profiling. Indeed, even in the most studied environmental niches such as HGM, taxonomic profiling of detected amplicon sequence variants (ASVs) provides only a partial strain-level resolution, with many ASVs having taxonomic descriptions at the species, genus, or even family level. It leads to a bioinformatic problem of accurate projection of the current knowledge on the presence/absence of functional features from reference genomes of individual strains to real-life ASVs (ensembles of taxonomically unresolved species and strains). Due to the intrinsic variations of many features within microheterogeneous ASVs, for computational purposes, such projection should be considered probabilistic and corresponding maps of ASVs to reference genomes should be accompanied by an estimated error reflecting the uncertainty due to these variations.

In previous studies, we have used a simple taxonomy-based mapping approach, which projects ASVs to the reference database of genomes with their respective binary phenotypes (termed *Binary Phenotype Matrix* or BPM) using a level-by-level comparison of the ASV’s taxonomic description with the taxonomies of the reference genomes. BPM entries with the best match were then taken to form the ASV-to-BPM map, with weights equally distributed across unique species. Furthermore, for each binary phenotype and ASV, a Phenotype Index (PI, a value between 0 and 1) was calculated as the map-weighted average binary phenotype of reference organisms across the ASV-to-BPM map. Each PI represents a probability for a given binary phenotype to be associated with the ASV and is accompanied by the corresponding prediction error due to an imprecise mapping. To improve the precision (minimize the uncertainty) of PI assignment to ASVs, we have introduced two additional mapping methods, the first of which relies on the so-called Multi-Taxonomic Assignment (MTA) scheme. In contrast to the commonly used Consensus-Based Taxonomic Assignment (CBTA) scheme [e.g., in consensus-blast plugin in QIIME2 (Bolyen et al., 2019)], where ambiguities are resolved by keeping taxonomic descriptions with sufficient consensus, MTA preserves multiple best-match species-level taxonomies in order to enhance taxonomic resolution. Alternatively, the second, sequence-based approach (SEQ) does not rely on intermediate taxonomies and aligns ASVs directly to the 16S reference database, thus, allowing to obtain a strain-level resolution for some ASVs. Here, we report the benchmarking of our phenotype prediction approach using all the above mapping schemes. For a dataset of 1,000 mock bacterial communities representing HGM with defined taxonomic composition and functional profiles, SEQ scheme demonstrated overall insignificant prediction uncertainties and, as anticipated, largely outperformed the CBTA and MTA schemes. The taxonomy-based schemes, however, showed a reasonable prediction accuracy for a subset of phylogenetically homogeneous phenotypes, thus justifying their use in the phenotypes-based analysis of taxonomic profiles in the absence of the 16S sequencing data (e.g., originating from whole genome shotgun sequencing).

Finally, a third critical aspect of functional bottom-up profiling methodology is the robust computational method of microbiome-wide aggregation of the functional traits of individual species into the community’s cumulative metrics, which should have an explicit biological interpretation. Such method would enable a computational comparative analysis of multiple HGM samples to support applications in diagnostics and rational development of dietary supplements for the prevention or correction of dysbiosis-related syndromes. A potential practical utility of such analysis based on binary phenotype encoding of metabolic properties can be illustrated by studies of defined microbial consortia in gnotobiotic mice model aimed at developing therapeutic food supplements for infants with dysbiosis triggered by malnutrition (Blanton et al., 2016; Gehrig et al., 2019; Raman et al., 2019).

Recently, we have introduced the computational approach to a microbiome-wide aggregation of metabolic properties assigned to ASVs (as outlined above) by calculating the Community Phenotype Indices (CPIs), i.e., abundance-weighted PIs. For a given phenotype, the CPI value corresponds to a fractional representation of the phenotype carriers in a microbiome sample. This simple metric was applied to the analysis of B-vitamin biosynthetic potential over large collections of 16S-profiled HGM samples from the Human Microbiome Project and American Gut Project studies (Human Microbiome Project Consortium, 2012; McDonald et al., 2018) and allowed us to detect a significant abundance of B-vitamins auxotrophs, in accordance with the micronutrient sharing hypothesis (Rodionov et al., 2019). This hypothesis was further supported by the studies in humanized gnotobiotic mice model and via anaerobic *in vitro* culturing in the context of extreme variations of B-vitamin supply (Sharma et al., 2019). The CPI-based functional profiling of HGM samples was applied to several other 16S rRNA metagenomic datasets, including the *in vitro* fermentation of fecal microbiomes (Peterson et al., 2019; Elmen et al., 2020) and the comparative analysis of metabolic properties in microbiomes of infants as a function of breast-feeding vs. formula (Jones et al., 2020), allowing us to link the metabolic phenotypes with variable environmental/growth conditions. Finally, predicted metabolic phenotypes were used for the classification of HGM samples from healthy versus IBD patients providing interpretable insights into the host-microbiome mechanisms of disease (Iablokov et al., 2021).

Here, we extended this bioinformatics approach for the metabolic phenotype profiling of HGM samples by incorporating novel metrics for a diversity-based description of the phenotype carriers, namely, Phenotype Alpha Diversity (PAD) and Phenotype Beta Diversity (PBD). These metrics were applied for the metabolic phenotype profiling of several large metagenomic datasets, with PAD serving as a measure of stability for a given functional trait (phenotype) and PBD being a promising method for identification of driving phenotypes.

## Materials and Methods

### Raw Data Analysis

Raw 16S rRNA gene sequencing data from two large metagenomic studies representing the general population, namely, from the American Gut Project (AGP) (McDonald et al., 2018) and the UK Twins study (UKT) (Goodrich et al., 2016), as well as from three IBD-related studies conducted in China (CHN) (Zhou et al., 2018), Spain (ESP) (Pascal et al., 2017), and Netherlands (NLD) for the IBD group (Imhann et al., 2018) and for healthy controls (Tigchelaar et al., 2015), were analyzed using the QIIME2’s dada2 plugin (Bolyen et al., 2019). 16S amplicons were quality-filtered and dereplicated into amplicon sequence variants (ASVs) with default parameters. Samples with reads counts below a certain threshold were discarded. For the AGP dataset, we additionally removed samples with high levels of blooms. Summary for the analyzed datasets, with read count thresholds and the remained number of samples, is presented in Table 1.

**TABLE 1:** Summary on the number of samples retained in each analyzed dataset after filtration by the minimum number of reads threshold.

Dataset	Number of samples	Min. number of reads
**AGP**	2,868	10,000
**UKT**	3,288	10,000
**CHN**	134 HC/75 CD	4,000
**ESP**	154 HC/140 CD	15,000
**NLD**	966 HC/163 CD	15,000

*For the IBD-related datasets (CHN, ESP, and NLD), the respective numbers are given for healthy (HC) and Crohn’s disease (CD) cohorts separately.*

### *In silico* Mock Communities

A set of 1,000 mock communities was randomly generated (*in silico*) from the reference collection of 2,662 bacterial HGM genomes (Rodionov et al., 2019; Iablokov et al., 2021) that are available in the SEED database (Overbeek et al., 2014). The number of unique species (S) for each community was sampled from the normal distribution (mean = 30, std = 5) with the restriction 10 < S < 60. Unique species names were then sampled from the list of 120 most abundant species in the UKT dataset, with weights equal to their mean abundance (A) across the dataset. For each unique species-level description, N organisms (strains) with the corresponding taxonomy were uniformly sampled from the reference HGM genome database. The number N was uniformly sampled between 1 and the ceiling value of the square root of the total number of organisms with the given species-level description. The respective relative abundance (R) of each species in a given mock community was sampled from a normal distribution (mean = A, std = A × 0.4) with the restriction A × 0.1 < R < A × 3. Values of R for each community were then rescaled to sum up to 1. The respective relative abundance fractions (Q) of strains within a species were uniformly sampled and rescaled to sum up to 1. Total amplicon count (T) for each community was sampled from a normal distribution (mean = 20,000; std = 4,000) with the restriction 4,000 < T < 40,000. Individual 16S amplicon counts (C) for the organisms (strains) in each community were then obtained as C = Q × R × T. These generated bacterial communities comprised the MOCK TRUE dataset with a confidently known one-to-one association (map) between each 16S sequence and the reference genome database. To model a real experiment, the corresponding 16S rRNA gene sequences were further truncated to the V3–V4 variable regions (flanked by 341F/806R primers). Truncated amplicons with identical sequences were collapsed into a single amplicon sequence variant (ASV) with aggregated abundance, comprising the MOCK AGGR dataset. The resulting ASV abundance tables and respective 16S sequences (for both the TRUE and AGGR mock datasets) are presented in Supplementary Table 1.

### Taxonomic Profiling and Abundance Renormalization

Taxonomic profiling of ASV representative sequences was performed following the Multi-Taxonomic Assignment (MTA) scheme. Specifically, 16S amplicons were aligned using NCBI BLAST+ (Camacho et al., 2009) against a joined reference 16S rRNA database with sequences from the RDP database version 11.5 (Cole et al., 2014) and NCBI 16S database version of December 2019. Alignment results were sorted according to the fraction (from 0 to 1) of their identity F, with the maximum *F* value for the alignment denoted as M. Top alignment hits with value of F in the range from M to M-(1-M)/S and a threshold greater than the value D were selected for MTA. Here, S acts as a scaling parameter, which controls the list of taxonomic descriptions accepted for MTA based on the *F* value of the alignment, and was taken equal to 4. The drop threshold parameter D limits the alignment quality from below, and was taken equal to 0.85. The strict choice of value for S is motivated by the necessity to investigate 16S sequences (and their corresponding organisms) in a small neighborhood of the top match, while keeping the MTA description compact. The value of D was chosen to discard the ASVs with a poor taxonomic resolution, for which metabolic phenotype predictions are highly inaccurate due to the high degree of phenotype heterogeneity within a broader phylogenetic group. The resulting multi-taxonomy for each ASV was a list of unique species-level taxonomic descriptions with equal weights assigned to each item. String representations for MTA consisted of slash-separated names of taxa on each taxonomic level, e.g., *Bacteroides ovatus/vulgatus* or *Escherichia coli/Salmonella enterica*.

Based on the MTA profile, we additionally performed a Consensus-Based Taxonomic Assignment (CBTA) by choosing 51%-consensus on each taxonomic level, leaving blank the assignment entries for taxonomic levels with an insufficient consensus.

Original dada2-derived 16S amplicon counts were further renormalized to account for the different 16S rRNA gene copy numbers (GCN) in microbial genomes. Average GCN values for taxonomic entities at different ranks were extracted from the rrndb-5.6 database (Stoddard et al., 2015) and mapped to ASVs using their MTA-based taxonomic descriptions. For each ASV, a simple mean GCN value was calculated and used as a factor to normalize the ASV’s abundance. Thus, obtained abundances were then re-scaled to sum up to 1.

### Binary Phenotypes for Reference Genomes

The comparative genome analysis and reconstruction of target metabolic pathways in the reference genome database containing 2,662 HGM genomes (representing ∼770 individual species) was previously conducted using the subsystems approach (Overbeek et al., 2005; Osterman et al., 2010) implemented in the SEED/RAST platform (Overbeek et al., 2014) as previously described (Rodionov et al., 2019; Iablokov et al., 2021). Briefly, the metabolic subsystems were manually built as groups of functional roles for enzymes, transporters, and transcriptional regulators that are involved in a specific aspect of the cellular machinery such as a metabolic pathway. Functional gene assignments and metabolic reconstructions were performed using the following three genome context techniques to functionally link a set of genes to a single pathway: (i) clustering of genes on the chromosome (operons), (ii) co-regulation of genes by a common regulator [regulons, as captured in the RegPrecise database (Novichkov et al., 2013)], and (iii) co-occurrence of genes in a set of related genomes (Overbeek et al., 2007). For the functional gene annotation and building metabolic subsystem in SEED, we combined the existing annotations with information from literature accessed via the PaperBLAST tool (Price and Arkin, 2017) and reference databases including the UniProtKB/Swiss-Prot for characterized proteins (Boutet et al., 2016), KEGG for reference metabolic pathways (Kanehisa et al., 2012), TCDB for transporter classification (Elbourne et al., 2017), and CAZy for classification of Glycosyl Hydrolases (Lombard et al., 2014).

Curated across HGM genomes metabolic subsystems include the central biochemical pathways classified into four categories: (i) biosynthesis of vitamins and cofactors, (ii) biosynthesis of protein-forming amino acids, (iii) utilization of carbohydrates and other carbon sources, and (iv) production of fermentation products including short-chain fatty acids (SCFAs) such as acetate, butyrate, and propionate. In many subsystems, we captured distinct biochemical pathway variants and numerous non-orthologous enzymes and transporters. Using pathway-specific logical rules that account for both the variable pathway and signature genes, we assigned binary phenotypes (1/0) for each metabolic phenotype and target genome. The resulting Binary Phenotype Matrix (BPM) contained 94 metabolic phenotypes reflecting the presence/absence of a complete catabolic or biosynthetic pathway. The following 24 representative phenotypes were a subject of analysis in this work (Figure 2): biosynthesis of B-vitamins (B1, B2, B3, B5, B6, B7, B9, B12), lipoate, and vitamin K; production of SCFAs (butyrate and propionate); utilization of carbohydrates (glucose, galactose, fructose, mannose, xylose, arabinose, fucose, rhamnose, ribose, lactose); and biosynthesis of amino acids (His, Trp). These phenotypes were chosen by a combination of criteria such as physiological relevance, extensive knowledge ensuring confidence of phenotype inference, and significant and variable representation across microbiome samples.

### ASV Mapping

To obtain the phenotype profiles for the analyzed 16S rRNA samples, we utilized a development version of the Phenotype Profiler tool provided by PhenoBiome Inc. (Walnut Creek, CA, United States^1^). Mapping of ASVs to the BPM was performed using three different schemes. Two of them are taxonomy-based (CBTA and MTA), and they use a level-by-level comparison of the ASV taxonomic descriptions with taxonomies of the reference genomes. Matches on the deepest taxonomic level were added to the ASV-to-BPM map with weights equally distributed (and summing up to 1) across unique species. Within each unique species, the weights were equally distributed between all strains. ASVs with a match on at least the family level were considered as mapped, with the rest being non-mapped. For multi-taxonomies, this procedure was applied for each simple taxonomic description in the MTA with additional weighting using the MTA weights. The third scheme is sequence-based (SEQ) and employs an ASV sequence alignment against the 16S database for reference genomes (from which phenotypes were derived). This process mirrors the MTA scheme with the same values for S and D parameters. ASVs with F values greater than D were considered as mapped. ASVs with all reference sequences having their *F* value below D were considered as non-mapped. The average values of ASV coverage by reference genomes, i.e., the abundance of mapped ASVs, for all analyzed datasets is presented in Table 2.

**TABLE 2:** Mapping coverage for the analyzed datasets.

Scheme	AGP	UKT	CHN	ESP	NLD
**CBTA**	81%	89%	–	–	–
**MTA**	84%	93%	–	–	–
**SEQ**	79%	86%	99%	97%	94%

*For each dataset (and mapping scheme where appropriate), the average abundance of ASVs mapped to the BPM is shown as percentage.*

### Assignment of Phenotype Indices

For each ASV and metabolic phenotype, we assigned a corresponding Phenotype Index (PI, from 0 to 1), representing the probability for the phenotype to be associated with the given ASV. PIs were calculated using the ASV-to-BPM map as the map-weighted averages of binary phenotypes from the BPM: *PI* = ∑*W*_*i*_×*P*_*i*_, where *W_i_* and *P_i_* are the mapping weight (from 0 to 1) and respective binary phenotype value (0 or 1) for the i^th^ organism in the ASV-to-BPM map. Assuming that the binary phenotypes follow the binomial distribution, we calculated variance of *Var*(*PI*) = *PI*×(1−*PI*), and have taken it as an estimate of PI prediction uncertainty. To measure the cumulative properties of microbial communities with respect to a given phenotype, we computed the Community Phenotype Indices CPIs as abundance-weighted average PIs for all phenotypes, *CPI* = ∑*A*_*i*_×*PI*_*i*_, where *A_i_* and *PI*_*i*_ are the relative abundance (from 0 to 1) and respective Phenotype Index (from 0 to 1) of the i^th^ ASV in the sample. Under the assumption of independent co-occurrence of ASVs, we calculated variance of CPI, $V\,a\,r\,\left(C\,P\,I\right)=\sum A_{i}^{2}\times V\,a\,r\,\left(P\,I_{i}\right)$, and have taken it as an estimate of CPI prediction error. The relative CPI prediction uncertainty (rSTD) was then calculated as the ratio of its standard deviation (square root of variance) and CPI itself. The computed CPI values for the *in silico* mock, AGP, and UKT datasets, as well as for the three IBD-related studies are provided in Supplementary Tables 2A,B, 7, respectively.

### Phenotype Diversity

The Phenotype Alpha Diversity (PAD) and Phenotype Beta Diversity (PBD) were estimated as, respectively, the alpha and beta diversity of the sub-communities of carriers of a particular phenotype. Briefly, a multiple alignment of ASV representative sequences was performed using MUSCLE (Edgar, 2004), followed by the construction of an unrooted phylogenetic tree with FastTree 2 (Price et al., 2010). The resulting tree was rooted according to the midpoint strategy and used (with ASV abundances and ASV-to-BPM map) in calculation of both PAD and PBD metrics with the Python’s scikit-bio package^2^. Sub-communities of the respective phenotype carriers were determined according to a PI > 0.6 threshold, with the abundances of selected ASVs normalized to 1 for each sample. Faith Phylogenetic Diversity (Faith, 1992) and Weighted UniFrac (Lozupone and Knight, 2005) were chosen as the respective alpha and beta diversity metrics. The computed PAD values for AGP and UKT datasets are provided in Supplementary Table 4.

## Results and Discussion

**FIGURE 1:**
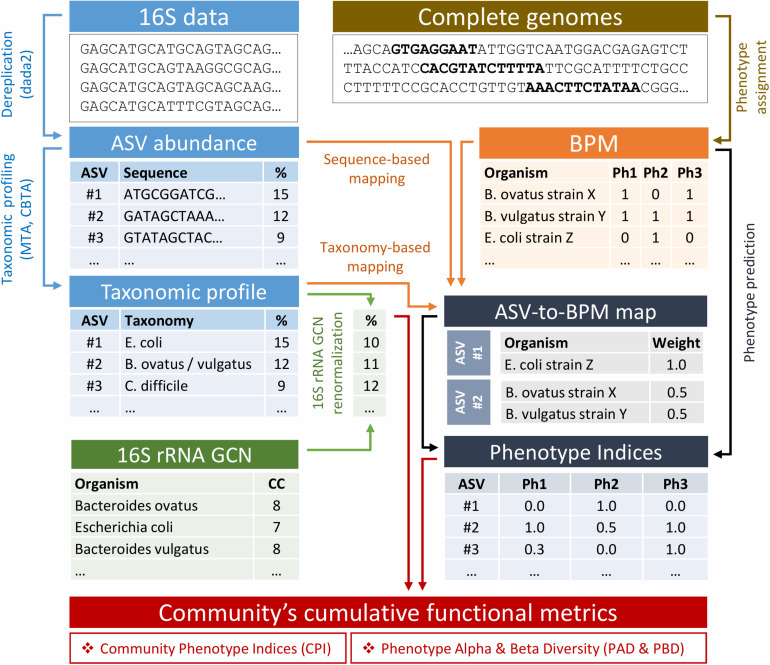
Flowchart of the phenotype profiler pipeline.

A computational pipeline for metabolic profiling of complex microbial communities represented by the 16S amplicon sequencing data is based on the probabilistic prediction of metabolic phenotypes for each ASV identified in the microbiome sample (see Methods). Thus, for each phenotype, an ASV is assigned a respective Phenotype Index (PI, from 0 to 1), i.e., a probability for the phenotype to be associated with a given ASV. The principal computational scheme of our pipeline is shown in Figure 1. It includes the following steps: (i) filtering of 16S rRNA amplicons and their dereplication into ASVs; (ii) taxonomic profiling; (iii) abundance renormalization due to variations in 16S rRNA gene copy numbers (GCN) among different taxa; (iv) mapping of ASVs to the reference genomes; (v) phenotype prediction; and (vi) calculation of community’s cumulative characteristics, including the Community Phenotype Index (CPI), Phenotype Alpha Diversity (PAD), and Phenotype Beta Diversity (PBD). For characteristics requiring phylogenetic data (PAD and PBD), the additional procedures of ASV multiple alignment and tree construction are implemented (not shown). In the following section, we assess the phenotype prediction method using the *in silico* generated mock communities with precisely known taxonomic composition and functional profiles and compare different approaches for the mapping of ASVs to the collection of reference genomes. Next, we discuss the potential application of the recently introduced Phenotype Alpha Diversity (PAD) metric as a measure of phenotype stability with respect to environmental change. Finally, we propose a similar concept of Phenotype Beta Diversity (PBD), which provides a diversity-based approach for the detection of metabolic features which are presumably associated with clinical status.

**FIGURE 2:**
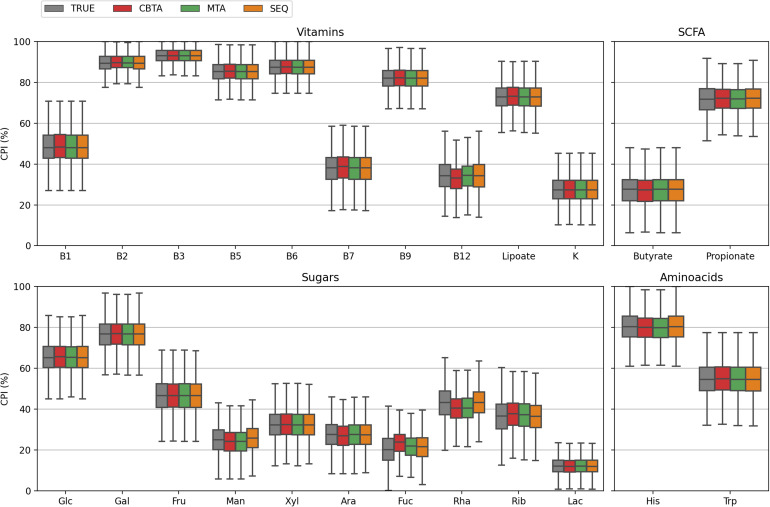
CPI value distributions for 1,000 *in silico* generated mock communities. Phenotype Indices (PI) are predicted according to four ASV-to-BPM mapping schemes: (i) TRUE, with true Pis; (ii) CBTA and MTA, with PIs predicted using taxonomy-based mapping schemes; (iii) SEQ, with PIs predicted using a sequence-based mapping scheme.

### ASV Mapping and Phenotype Prediction

Throughout previous studies, we used three different schemes for the prediction of phenotypes, one scheme being an upgrade of another. Two of them employ taxonomic assignments to map ASVs to reference genomes (ASV-to-BPM map), with the level-by-level comparison of respective taxonomic strings (see Methods). The naivest scheme makes use of the Consensus-Based Taxonomic Assignment (CBTA) approach, which resolves ambiguities by keeping the descriptions only for the taxonomic levels with a sufficient consensus. A somewhat advanced scheme is based on the Multi-Taxonomic Assignment (MTA) approach, which retains all relevant simple taxonomic descriptions equally weighted, with string representations for MTA consisting of slash-separated taxonomies, e.g., *Bacteroides ovatus/vulgatus* or *Escherichia coli/Salmonella enterica*. Lastly, the third scheme utilizes the sequence-based (SEQ) mapping approach, which relies on the alignment of ASVs directly against the 16S rRNA sequences of the reference organisms.

**FIGURE 3:**
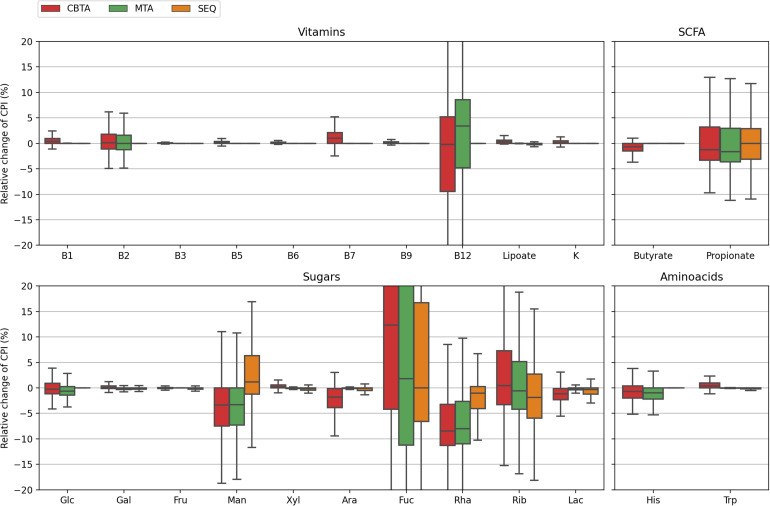
Distributions of the relative change in the CPI values for 1,000 *in silico* generated mock communities. CPI values are calculated using three ASV-to-BPM mapping schemes (CBTA, MTA, SEQ) and compared with true CPIs.

In this study, we assessed the performance of our computational approach by analyzing the accuracy of phenotype prediction for 1,000 *in silico* generated mock bacterial communities representing HGM with defined taxonomic composition and functional profiles. We calculated the Community Phenotype Indices, i.e., abundance-weighted PIs, and relative CPI prediction uncertainties (see Methods) for the three proposed ASV mapping schemes (CBTA, MTA, and SEQ) as well as for the true mapping scheme (TRUE) known upon the generation of mock communities (Supplementary Table 2A). The obtained CPI distributions (Figure 2) demonstrated a high degree of similarity, therefore, making a dataset-wise description of functional traits independent of the mapping scheme. From a sample-by-sample comparison of the predicted CPIs with their true values (Figure 3), it is apparent that the SEQ mapping scheme significantly outperforms (Supplementary Table 3) the taxonomy-based schemes (CBTA and MTA). This is somewhat an expected result, because the V3–V4 variable regions of the 16S rRNA gene allows for a partial strain-level taxonomic resolution, thus, decreasing the phenotype prediction error. Among the latter two schemes, MTA showed overall lower values in the relative change of CPI, most likely due to the phenotype averaging across a phylogenetically narrower group of organisms as compared to CBTA.

**FIGURE 4:**
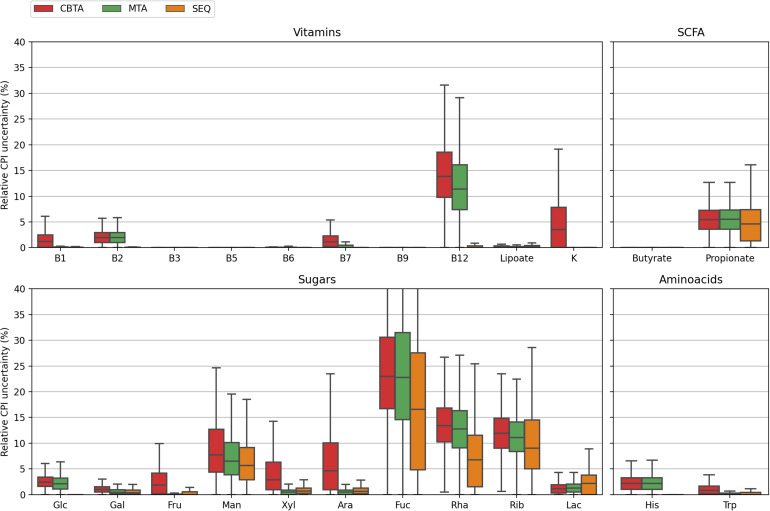
Distributions of the relative CPI prediction uncertainties calculated for 1,000 *in silico* generated mock communities using three mapping schemes.

Despite the generally poorer performance of the taxonomy-based mapping approaches, they nonetheless demonstrated a reasonable phenotype prediction accuracy for the majority of the considered phenotypes, with typical discrepancies from the true values of the order of 5%. This suggests the potential use of the taxonomy-based mapping for functional profiling of metagenomic samples lacking a 16S sequencing data, however, described by taxonomic profiles derived from, e.g., shotgun whole-metagenomic sequencing. This seems especially promising for the profiling of highly conservative phenotypes (such as B-vitamin or amino acid biosynthesis), i.e., functional traits with less variability in taxonomically close microbial genomes. Another important observation is that for some phenotypes, namely, fucose degradation (Fuc) and ribose degradation (Rib), even the SEQ mapping scheme demonstrated inaccurate phenotype predictions of the order of 10% and larger. This is due to the high degree of their phylogenetic microheterogeneity which is straightforwardly observed when considering the corresponding distributions for the relative CPI prediction uncertainties (Figure 4). Unlike the genuine prediction errors (Figure 3) estimated with respect to the true CPI values of mock communities, the relative CPI uncertainties were calculated based solely on the ASV mapping schemes (CBTA, MTA, and SEQ). The observed correlation between the true phenotype prediction errors and relative CPI uncertainties strongly suggests the use of the latter as a measure of phenotype prediction ignorance.

**FIGURE 5:**
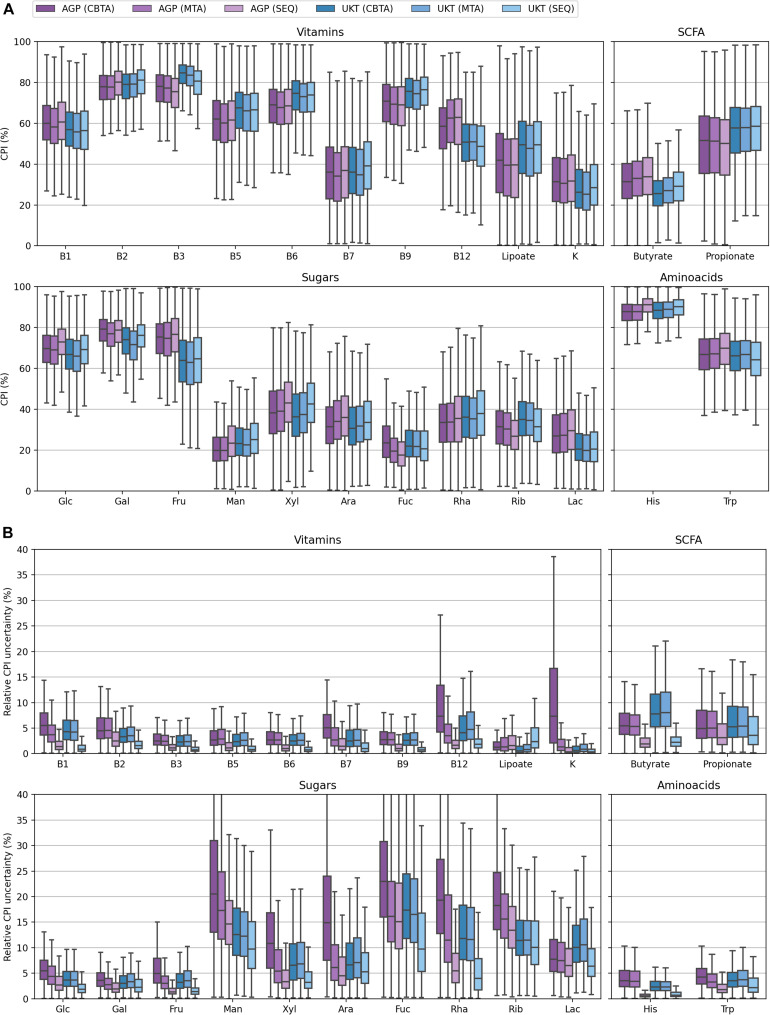
Distributions of CPI values **(A)** and their relative uncertainty values **(B)** for the AGP and UKT datasets calculated using three ASV-to-BPM mapping schemes.

To assess the performance of our computational approach for functional profiling of complex microbial communities on real metagenomic data, we analyzed gut samples from two large HGM datasets, namely the American Gut Project (AGP) and UK Twins study (UKT). We calculated the respective CPI values (Figure 5A) and corresponding relative CPI prediction uncertainties (Figure 5B) using three ASV-to-BPM mapping schemes, namely, CBTA, MTA, and SEQ (Supplementary Table 2B). The resulting CPI distributions showed a striking similarity between these mapping schemes for both datasets, which is in good agreement with our previous discussion. The magnitudes of relative CPI prediction uncertainty for both datasets mirror those observed for the mock communities, with the SEQ mapping scheme demonstrating an overall greater performance. The only minor exception is that of small, however, non-zero, values of the relative CPI prediction uncertainty, that were measured in both datasets even for the phenotypes with a high level of phylogenetic homogeneity. These uncertainties are driven by the presence of taxa, which are poorly covered by reference genomes, thus, leading to the ambiguous ASV-to-BPM maps. Finally, the overall level of CPI prediction uncertainty was usually higher for the AGP samples than for the UKT samples, which is due to the lower length of the sequenced 16S rRNA gene region in the AGP dataset (150 nts) as compared to the UKT study (292 nts), thus, explaining the superior taxonomic resolution in the latter case.

In summary, both taxonomy-based (CBTA and MTA) and sequence-based (SEQ) mapping schemes demonstrated a reasonable phenotype prediction accuracy for the majority of the metabolic phenotypes in both *in silico* generated mock communities and samples from large HGM studies (AGP and UKT), with the SEQ mapping scheme significantly outperforming the taxonomy-based approaches. It is expected that the use of full-length 16S amplicons will further increase the prediction accuracy of SEQ, thus, allowing for the reliable profiling of metabolic phenotypes even with a high degree of phylogenetic microheterogeneity. In further analysis, the use of the SEQ mapping scheme is assumed.

### Phenotype Alpha Diversity

**FIGURE 6:**
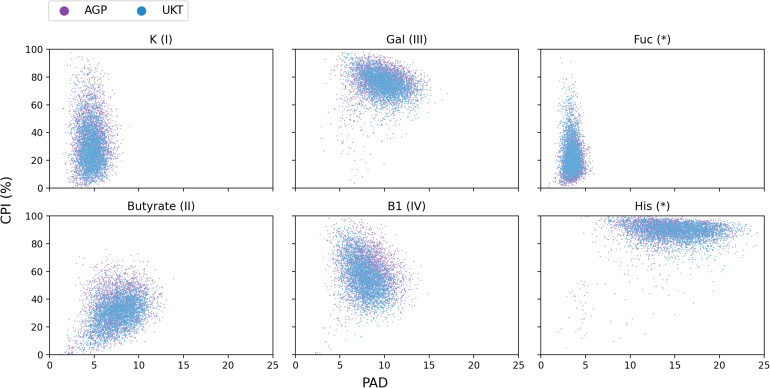
CPI-vs.-PAD scatterplots for six selected metabolic phenotypes calculated for the AGP and UKT datasets.

In a recent paper (Iablokov et al., 2021), we introduced a concept of Phenotype Alpha Diversity (PAD), which serves to describe the alpha diversity for a sub-community of carriers of a particular phenotype, thus, reflecting how phylogenetically broad or narrow this sub-community is. To further develop this concept, we computed the PAD values for the AGP and UKT datasets (Supplementary Table 4) and investigated the CPI-vs.-PAD relationship. The respective scatterplots are shown in Figure 6 for six selected phenotypes and in Supplementary Figure 1 for the rest of the phenotypes. Based on a set of descriptive statistics for the CPI and PAD distributions (Supplementary Table 5), we clustered 22 phenotypes into four categories according to the shapes of their respective CPI-vs.-PAD clouds, described by median PAD values (PAD Q50), median CPI values (CPI Q50), and 10–90 percentile range (10–90 PR) of CPI variation (Table 3). The remaining two phenotypes (Fuc and His) remained unclassified since they demonstrated marginal features.

**TABLE 3:** Phenotype categories and their observed characteristics.

Category	I	II	III	IV
**Phenotypes**	B7, Lipoate, K, Xyl, Ara, Rha, Propionate	Man, Rib, Lac, Butyrate	B2, B3, B6, B9, Glc, Gal, Trp	B1, B5, B12, Fru
**CPI variation**	Moderate to large (35.6–49.5)	Moderate (27.7–33.0)	Moderate to small (21.4–32.5)	Moderate (35.1–40.1)
**Median PAD**	Small (3.3–6.4)	Small to moderate (5.1–7.6)	Large (9.8–11.4)	Moderate to large (7.9–10.2)

**FIGURE 7:**
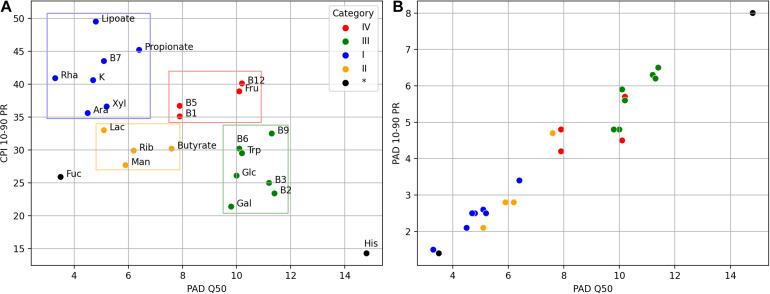
Mutual dependence of the CPI and PAD values obtained for the AGP and UKT datasets. **(A)** Dependence between median PAD (PAD Q50) and variation of CPI (CPI 10–90 percentile range). A total 22 metabolic phenotypes are clustered into four categories, with two phenotypes remained unclassified. **(B)** Dependence between median PAD (PAD Q50) and variation of PAD (PAD 10–90 percentile range).

The CPI and PAD metrics allow us to relate the variation of phenotype abundance with its diversity, the latter likely accounting for the phenotype stability in the face of environmental perturbations. For phenotypes with a generally low PAD (e.g., as in categories I and II), one expects moderate to large variations in the respective CPI values due to taxonomic shifts caused by such environmental changes. At the same time, well-diversified phenotypes (such as in category III and His) are expected to demonstrate large median CPI values, leaving little room for a significant CPI variation. This hypothesis is indeed supported by the overall decreasing trend in the CPI 10-90 PR vs. median PAD plot (Figure 7A) for the analyzed phenotypes.

We also notice that phenotypes with large PAD values (categories III and IV) have a strong tendency to show large PAD variations (Figure 7B) across the analyzed samples. Thus, for these phenotypes, PAD itself can be used as a non-redundant description of microbial communities. However, this is not entirely true for the histidine biosynthesis (His) phenotype. Despite the great variation in its PAD values (10–90 PR = 8), Phenotype Alpha Diversity for the His phenotype becomes a less efficient metabolism-driven description due to its significant correlation with the total alpha diversity. This conclusion should also hold for the majority of other amino acid biosynthesis phenotypes, which are even more abundant than His. Lastly, the fucose utilization (Fuc) phenotype has both a low diversity (PAD Q50 = 3.5) and abundance (CPI Q50 = 19.3). It also demonstrates an exceptionally low CPI variation (10–90 PR = 25.9), thus, not entirely following the “low diversity–high variation” pattern observed for other phenotypes. This is probably due to the fact that fucose is a rare monosaccharide, which is present as a minor constituent in host-derived glycans such as mucin (Tailford et al., 2015) and human milk oligosaccharides (Bode, 2009), and fucose utilization represents a somewhat rare functional capability among HGM bacteria. Similar PAD vs. CPI dependence is expected for other phenotypes describing the utilization of rare carbohydrates (data not shown).

Overall, Phenotype Alpha Diversity provides a measure of diversity for the sub-communities of phenotype carriers, similar to the functional redundancy index (FRI) used in Tax4Fun2 (Wemheuer et al., 2020). Despite the conceptual resemblance with PAD, the latter employs data on gene families [such as from the KEGG database (Kanehisa et al., 2012)], while in the present computational approach, the considered functional traits are the actual biological phenotypes, e.g., a capability for a vitamin biosynthesis or a sugar utilization. It should be noted that the PAD metric has an important application for sample classification tasks. For classifiers with phenotype metrics (such as CPI) used as features, PAD can serve as a filtering criterion, allowing one to discard phenotypes with insufficient diversity of carriers across the analyzed samples. We successfully applied this approach to construct classifiers for healthy vs. Crohn’s disease subjects with only phylogenetically well-diversified phenotypes (Iablokov et al., 2021). This permitted us to interpret the classification outcome in a truly metabolism-driven manner, i.e., in terms of potentially driving phenotypes.

### Phenotype Beta Diversity

In a complete analogy to Phenotype Alpha Diversity, we introduce the concept of Phenotype Beta Diversity (PBD) as the beta diversity (i.e., distances between samples) for the sub-communities of phenotype carriers. To show the potential applications of PBD, we analyzed gut samples in the healthy (HC) and Crohn’s disease (CD) groups from three inflammatory bowel disease (IBD) studies conducted in China (CHN), Spain (ESP), and Netherlands (NLD). The respective PBD values for the analyzed phenotypes, as well as the total beta diversity (BD), were calculated using the Weighted UniFrac beta diversity metric. To estimate the intragroup similarity between samples within the HC and CD groups, we computed the respective mean pairwise distances using both BD distance matrix and PBD distance matrices for each phenotype (Supplementary Table 6). To account for the inheritance of the diversity scale by the phenotype carriers’ sub-community from the total community, we also calculated the relative PBD (rPBD) as the ratio of the PBD value for a given phenotype over the total BD, for each of the HC and CD groups.

The values of rPBD that are close to 1.0 correspond to the same level of dissimilarity between samples for either of the two approaches, BD or PBD. Any deviation from 1.0 serves as an indicator for an increase or decrease in dissimilarity when passing from the total microbial communities to the sub-communities of phenotype carriers. Among all analyzed phenotypes, the butyrate synthesis (Butyrate), lactose (Lac) degradation, and B12 vitamin synthesis (B12) demonstrated a significant drop in both PBD and rPBD values for the HC samples when compared with the CD samples (Tables 4A–C). This observation suggests that in healthy subjects, the sub-communities of each of the above phenotype carriers are more similar to one another than the respective sub-communities in Crohn’s disease patients, as if following the famous “Anna Karenina” principle for microbiomes. This principle states that gut bacterial communities of healthy people are alike, while disease-associated microbiomes are different in their own way. Notably, here, this principle is valid not for the entire microbial communities but rather for the sub-communities of Butyrate-, Lac-, and B12- phenotype carriers.

**TABLE 4:** Intragroup similarity of carriers of selected three metabolic phenotypes in the HC and CD groups.

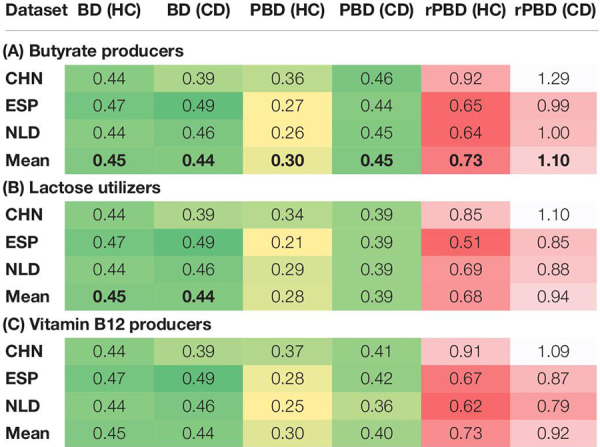

*Pairwise mean values for total beta diversity (BD), Phenotype Beta Diversity (PBD), and relative Phenotype Beta Diversity (rPBD) are shown for each IBD dataset for both healthy (HC) and Crohn’s disease (CD) groups. Bold font was used to emphasize the last row with mean values. Coloring of cells reflects the corresponding magnitudes in order to facilitate visual perception.*

**FIGURE 8:**
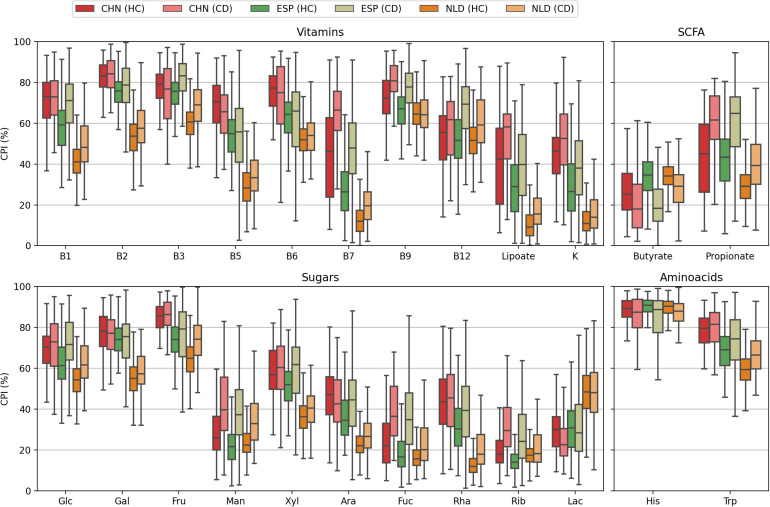
Distributions of the CPI values calculated for samples from heathy (HC) and Crohn’s diseases (CD) cohorts in the CHN, ESP, and NLS datasets.

Remarkably, the level of PBD-dissimilarity between samples within the HC and CD groups is not explicitly associated with the corresponding differences in CPI (Figure 8 and Supplementary Table 7). For Butyrate, the mean CPI is greater in the HC group for all datasets. The converse is true for B12, while for the Lac phenotype, there is no obvious pattern. Moreover, for other phenotypes with significant CPI differences between the HC and CD groups in all datasets (such as for B7, Lipoate, Propionate, Man, Fuc), almost identical levels of intragroup PBD-dissimilarity are observed (Supplementary Table 6). These evidences suggest that Phenotype Beta Diversity acts as another complimentary (to CPI) dataset-wise description of microbial communities. PBD accounts for the phylogenetic data and provides a diversity-based approach for the detection of metabolic features that are presumably associated with clinical status.

## Conclusion

In this study, we further developed our computational approach for the predictive functional profiling of complex microbial communities, which is based on the concept of binary metabolic phenotypes. Phenotype prediction accuracy was assessed using both (i) the *in silico* generated mock bacterial communities representing HGM with defined taxonomic composition and functional profiles, and (ii) two large metagenomic HGM datasets. The sequence-based scheme for ASV mapping to reference genomes demonstrated overall insignificant prediction uncertainties and outperformed the taxonomy-based mapping schemes. However, for phenotypes which are largely conserved at least on the level of species (such as B-vitamin synthesis), even the taxonomy-based predictions were of reasonable accuracy. It suggests the applicability of our approach for the metabolic profiling of samples that lack a 16S sequencing data and that are described by taxonomic profiles (e.g., originating from shotgun metagenomic sequencing or qPCR). In addition to the abundance-based description of functional traits (phenotypes) in terms of their Community Phenotype Indices, we also considered two diversity-based metrics, Phenotype Alpha Diversity and Phenotype Beta Diversity, that describe the diversity of sub-communities of phenotype carriers. Overall, greater variations of CPI were observed for phenotypes with a low PAD and vice versa, phenotypes with large PAD values demonstrated moderate to low variation of CPI. This makes PAD likely accounting for the phenotype stability in the face of environmental perturbations. Being also a useful criterion for the selection of phylogenetically well-diversified phenotypes for classification tasks (Iablokov et al., 2021), PAD metric itself represents a complementary (to CPI) description of microbial communities, and, when used as feature, is expected to improve the performance of classification and provide additional insights based on phenotype diversity. The PBD metric introduced in this study was used in the comparative analysis of HGM samples from healthy vs. Crohn’s disease cohorts. Notably, PBD values for a subset of phenotypes (Butyrate, Lac, B12) were much lower for the healthy subjects as compared to Crohn’s disease patients. This illustrates a potential diagnostic utility of PBD metric for a diversity-based detection of metabolic features associated with a particular syndrome. Both phenotype diversity metrics (PAD and PBD) can be also adopted to the sub-communities of phenotype non-carriers such as in the analysis of auxotrophy for essential nutrients (e.g., B-vitamins).

## Data Availability Statement

Publicly available datasets were analyzed in this study. This data can be found here: www.ebi.ac.uk/ena, project IDs PRJEB11419 (AGP), PRJEB13747 (UKT), PRJNA422193 (ESP), and PRJEB22028 (CHN); ega-archive.org, project IDs EGAS00001002702 (NLD IBD) and EGAS00001001704 (NLD Healthy, LifeLines DEEP).

## Author Contributions

DR, PN, and SI conceived and designed the research project. SI performed the primary analysis of the sequencing data. SI and DR performed the phenotype profiling. SI, DR, and AO wrote the manuscript. All authors developed the CPI/MTA/PAD/PBD concepts, read, and approved the final manuscript.

## Conflict of Interest

PN, DR, and AO are co-founders of PhenoBiome Inc., a company pursuing development and biomedical applications of computational tools for predictive phenotype profiling of microbial communities. The remaining author declares that the research was conducted in the absence of any commercial or financial relationships that could be construed as a potential conflict of interest.

## Footnotes

1 www.phenobiome.com

2 http://scikit-bio.org
