# Supplementary material for: Binary Metabolic Phenotypes and Phenotype Diversity Metrics for the Functional Characterization of Microbial Communities
Source: Front Microbiol. 2021 May 25;12:653314. doi: 10.3389/fmicb.2021.653314 (PMC8185038; doi:10.3389/fmicb.2021.653314)
Supplement: Supplementary file 1 [file Data_Sheet_1.PDF]

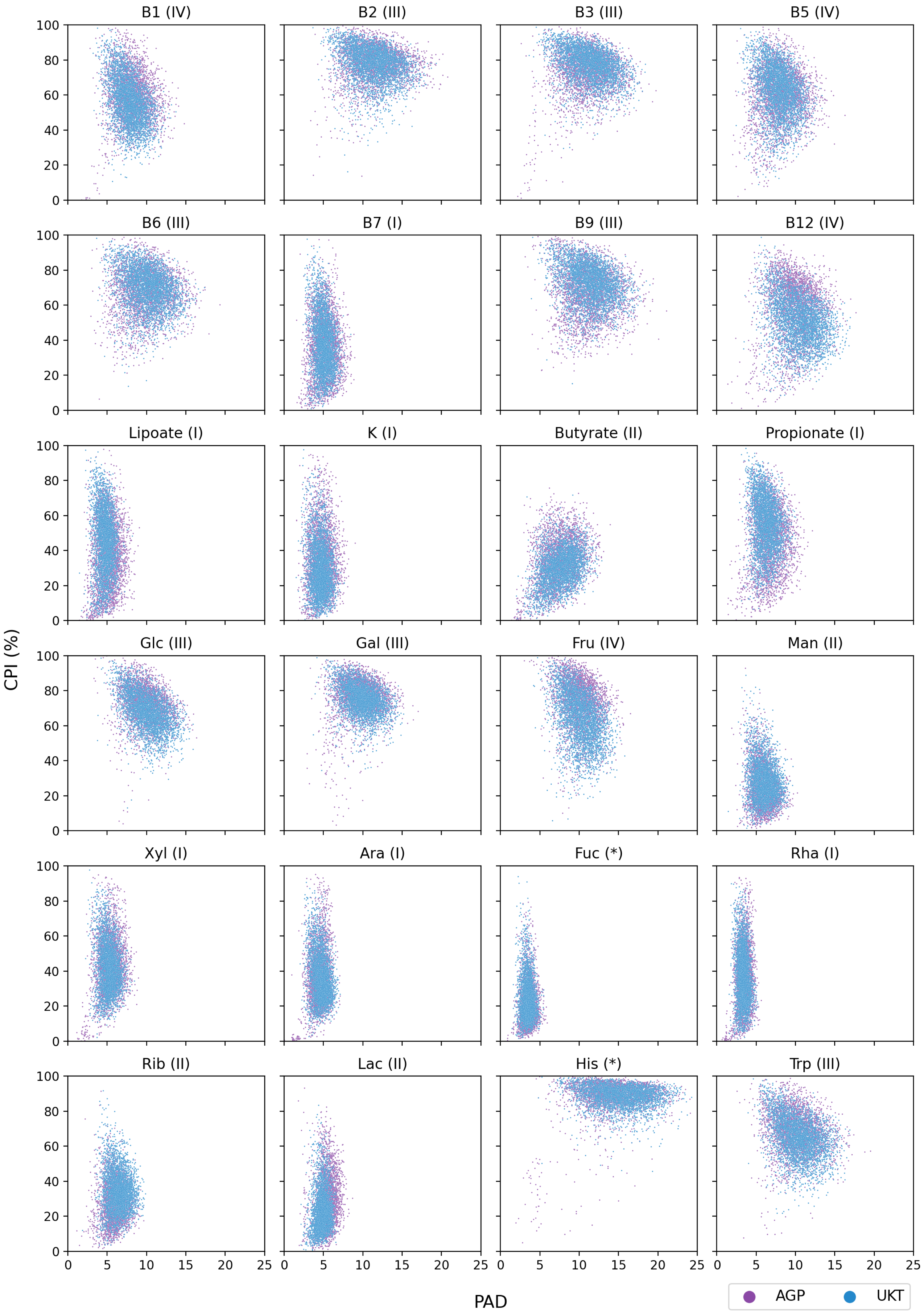

Supplementary Figure S1. The CPI-vs-PAD scatterplots for AGP and UKT datasets for 24 metabolic phenotypes. 22 phenotypes are clustered into 4 categories (I, II, II, and IV) and the remaining two phenotypes (His and Fuc) left unclassified (*).
